# A close relationship between HIF-1α expression and bone metastases in advanced NSCLC, a retrospective analysis

**DOI:** 10.18632/oncotarget.27378

**Published:** 2019-12-17

**Authors:** Aldo Pezzuto, Giuseppe Perrone, Nicoletta Orlando, Fabrizio Citarella, Massimo Ciccozzi, Simone Scarlata, Giuseppe Tonini

**Affiliations:** ^1^ Cardiovascular-Respiratory Science Department, Sant’ Andrea Hospital-Sapienza University, Rome, Italy; ^2^ Department of Pathology, Policlinico Campus Bio-Medico di Roma, Rome, Italy; ^3^ Oncology Department, Policlinico Campus Bio-Medico di Roma, Rome, Italy; ^4^ Department of Medical Statistics and Molecular Epidemiology, University Campus Bio-Medico, Rome, Italy; ^5^ Department of Internal Medicine, University Campus Bio-Medico, Rome, Italy

**Keywords:** HIF-1α expression, lung cancer, bone metastases, time to progression

## Abstract

**Background:** Hypoxia-inducible factor (HIF-1) is a transcription factor produced in hypoxia condition, it is closely associated with tumor angiogenesis and metastasis.

**Aim:** To investigate the expression of HIF-1α in relation with the presence or absence of bone metastasis. Methods A retrospective analysis was carried out on samples deriving from bronchial biopsy and CT-guided trans-thoracic needle biopsy. Detection of HIF-1 expression was performed on tissue sample by a monoclonal murine antibody, comparing patients with or without bone metastases (BM+).

**Findings:** In the total population the main histotype was adenocarcinoma (71.5%), COPD the prevalent comorbidity (73.6%), the mean pack-year was 36.4. Ninety-five histology samples were considered for analysis and comparison. Subdividing the population according to the presence or not of bone metastases, significant differences were found in pack-years (*p* = 0.02), time to progression (TTP) (*p* = 0.001) and COPD comorbidity (*p* = 0.04). The survival comparison between the two subgroups obtained by Kaplan–Meier method showed a longer TTP in patients with visceral metastases with a HR of 1.3 though the comparison by this method was not significant (*p* = 0.1). A higher intensity and percentage of expression of HIF-1α was recorded in the group with bone metastases (*p* = 0.02). The main variable affecting HIF expression in a multivariate analysis was the presence of bone metastases (*p* = 0.01).

**Interpretation:** Patients affected by NSCLC IV stage with bone metastasis have lower survival. There is a very close link between bone metastasis and HIF-1α expression level. The latter could be considered a predictive factor of bone spread and poor prognosis.

## INTRODUCTION

Hypoxia is a frequent condition detectable in roughly 50% of solid tumors owing to high proliferation rate of cancer cells along with altered vascularization [1]. The hypoxic tumor microenvironment influences both early and late stage of the disease. HIF-1α is a protein ubiquitously expressed and notably produced by tumor cells in hypoxic condition. It is a heterodimer helix-loop protein with a carboxy- and amino-terminus consisting in form α and β and it is the oxygen homeostasis master regulator binding to hypoxia responsive element (HRE) on target genes. The form β is constitutively expressed within the nucleus, by contrast the form α is expressed in oxygen-dependent manner and present in the cytoplasm [1].

HIF-1α is associated with cell activation, metastasis occurrence, and resistance to chemotherapy [2, 3]. In normal oxygen supply conditions the aforementioned factor is located in the cytoplasm and hydroxylation of proline residues occurs by means of hydroxylase enzymes (EGLN) that in turn allows von Hippel Lindau tumor suppressor (VHL) to bind to HIF and to elicit protein degradation by the ubiquitin proteasome system [4]. Conversely in hypoxic conditions, HIF-1α is unable to bind to the VHL protein that subsequently escapes decomposition and enters the nucleus. In the nucleus, it combines with HIF-1β to form the HIF-1 stable complex, which binds to DNA and acts as a transcription factor. HIF-1 brings to the activation of genes involved in angiogenesis, glycolysis, cancer proliferation and other associated pathways [5].

The HIF-1 is involved in neo-angiogenesis and in bone metastasis mechanisms, and it is able to elicit the expression of growth factors such as VEGF. *In-vitro* and animal model showed that hypoxia and HIF-1 expression contribute both to bone loss paving the way development of bone metastases [6, 7].

There is a strong link between hypoxia, HIF-1 expression and smoking habit. In a rat model for COPD, using exposure to LPS and cigarette smoke it was shown that expression of hypoxia inducible factor 1a gene was increased [8].

Indeed, the oncogenic role of cigarette smoking is promoted by benzopyrene, an aromatic policyclic hydrocarbon (PAH) able to activate the receptor for the epidermal growth factor receptor (EGFR) and then cell proliferation [9].

Cigarette smoke is also responsible for the dysfunction of bone metabolism through several mechanisms such as intestinal calcium absorption and sex hormone production. It also favors bone metastases through the activation of several growth factors, transcriptional factors, oncogene activation and inhibition of apoptosis [10, 11]. It is responsible of about 80% of lung cancer development.

We know that lung cancer is the leading cause of cancer-related death worldwide. Non-small cell lung cancer (NSCLC) encompasses about 80% of all lung malignancies. More than half of NSCLC patients are diagnosed when tumor is at a late stage (III B and IV) and the only option is systemic chemotherapy [12, 13].

However, 5-year survival rate of these patients remains below 10% in patients without activating EGFR or ALK mutation [14].

Tumor metastases is a major challenge issue, responsible for cancer cell death, frequently occurring in visceral and bone sites [7].

Our search was focused on the potential prognostic role of hypoxia-related HIF-1α in bone metastatic non small cell lung cancer.

### Aim of the study

The HIF-1α expression was already studied *in vitro* and *in vivo* in lung cancer and it is associated with poor prognosis.

The aim of the present manuscript is to better understand the association between HIF-1 α and lung cancer bone metastases and its influence on prognosis.

### Hypothesis

HIF-1α is higher expressed in patients affected by lung cancer with bone metastases than in patients without it.

### Primary endpoint

To determine the expression of HIF-1α in patients suffering from metastatic NSCLC comparing the group with bone metastases with the group without bone metastases.

### Secondary endpoint

To correlate the expression of HIF with smoking status, bone metastases and prognosis and to determine differences in terms of time to progression (TTP) between the group with bone metastases and the group without bone metastases.

## RESULTS

We focused on 95 patients among 146 who had an histology sample positive for primary non small cell lung cancer coming from trans-thoracic biopsies. Sixty-one of 95 histology samples were eventually available for HIF detection. Table 1 summarizes the characteristics of the histology samples: 95 patients Mean age was 72.6 ± 8.7, range 48-88, the ECOG PS was 1 for about 78% of patients.

**Table 1:** Overall population with histological sample: trans-thoracic biopsy specimens (84) + bronchial biopsy (11)

**Total 95 histology samples**	
Age	
Mean ± SD	72.6 ± 8.7
range	48–88
Gender M/F	62/33
Histotype	
Squamous	17 (17.8%)
large cell	7 (7.3%)
undifferentiated	3 (3.1%)
Adenocarcinoma	68 (71.5%)
PS ECOG 1	75 (78.9%)
COPD	70 (73.6%)
Bone Metastases + Visceral Metastases	40 (42.1%)
Visceral Metastases	55 (57.8%)
Smoking status	
former smoker	71 (74.7%)
current smokers	14 (15.0%)
non smokers	10 (10.5%)
mean pack year smoking	35.7 ± 12.5
Main Comorbidities: hypertension	45 (30.8%)

The histotype frequency was: 17.8% squamous, adenocarcinoma 71.5%, large cell carcinoma 7.3%, undifferentiated 3.1%.

Forty patients (42.1%) presented with visceral and bone metastases 10 of which reported bone metastases only. The remaining 55 (57.8%) had only visceral ones.

The main comorbidity was COPD present in 70 (73.6%) of patients.

The smoking status was the following: mean pack-year 35.7, former smokers were 71 (74.7%), current smokes were 14 (15.0%), non smokers only 10 (10.5%).

A comparison between patients having or not bone metastases is displayed in Table 2 including only trans-thoracic biopsy samples (95 patients).

**Table 2:** Comparison between groups: 95 histology specimens

	Bone metastases	Visceral metastases	*p*
Pack-year	27.5 (25.0–35.0)	37.5 (30.0–50.0)	0.02°
Age	72.0 (68.0–80.0)	73.0 (67.0–77.5)	0.28°
ECOG PS	1.0 (1.0–2.0)	1.0 (0.8–1.5)	0.10°
Further cancer%	8.2	17.0	0.04°°
TTP	6.0 (4.0–7.5)	7.0 (6.0–9.0)	0.001°
Adenocarcinoma%	76.0	75.0	0.14°°
Heart comorbidity%	24.0	45.0	0.06°°
COPD%	27.0	51.0	0.04°°
EGFR mutation%	8.0	11.0	0.15°°

Mann–Whitney test° and Fisher’s exact test.°°

Values are expressed as median and interquartile range (IQR) for Mann–Whitney test.

There was a significant difference (*p* = 0.02) in pack-years between patients with visceral metastases and patients with also BM. A difference was also reported by Mann–Whitney test in terms of TTP with median of 7 months for visceral metastatic versus 6 months for bone metastatic group (*p* = 0.001), and COPD comorbidity (*p* = 0.04) which was more frequent in visceral metastatic group. No significant difference were found about age, ECOG PS, heart comorbidity, EGFR mutation frequency.

**Figure 1:**
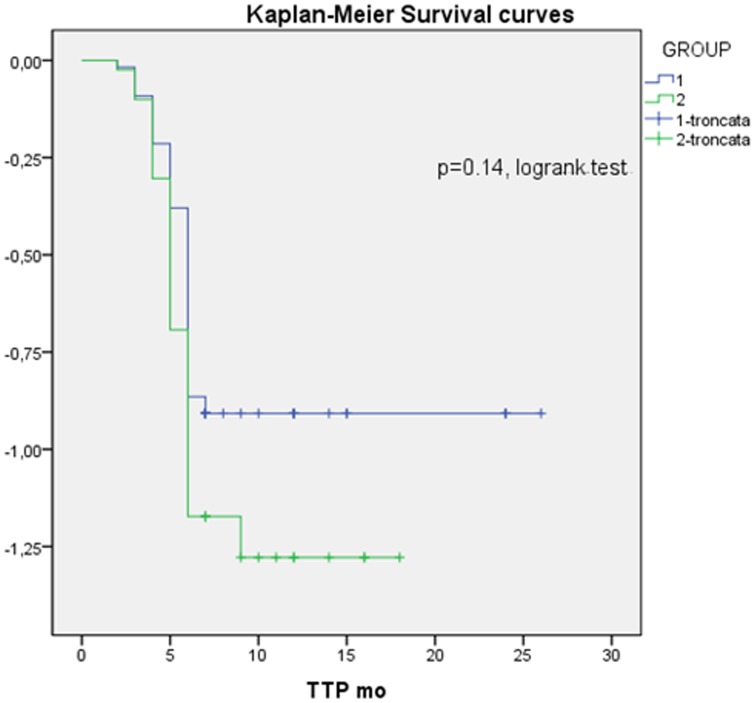
Kaplan-Meier survival curve; HR: 1.3. 95% CI: 0-8-2.2, Group 1: without bone metastases; Group 2: with bone metastases.

In Figure 1 the difference between groups in terms of TTP curves was also analyzed by Kaplan-Meier test, is shown with a HR 1.3 (IC 0.8–2.2); the data were not significant using that test but there was a trend of better course of disease in group without bone metastases with a longer time to progression. It happens because the log rank is a test that considers the data homogeneously along the curve while at the beginning the two curves cross, so it is a representative test but may not give the significance compared with Mann–Whitney test.

The influence of variables that were positive at univariate analysis on HIF-1 α is represented in Table 3 in a multivariate regression logistic analysis, highlighting that bone metastases significantly affect HIF-1 expression.

**Table 3:** Influence of variables on HIF expression

	OR	CI	*p*
Pack-Year	0.96	0.91–1.05	0.50
Age	0.95	0.85–1.04	0.23
ECOG PS	2.93	0.62–14.21	0.18
Bone metastases	8.04	1.42–44.10	0.01
Histotype	0.94	0.81–7.82	0.11

Thus the HIF expression is significantly higher in presence of bone metastases (*p* = 0.01).

Other parameters did not affect significantly HIF-1 α expression level. In Table 4 the influence of different parameters on TTP was analyzed in a multivariate model by cox-proportional regression method. The histotype (adenocarcinoma/squamous) was shown to be the only variable affecting significantly the time to progression, increasing the probability of time to progression at six month (*p* = 0.04).

**Table 4:** Influence of variables on TTP

Covariate	HR	CI	*p*
Pack-Year	0.99	0.96–1.02	0.64
Age	1.00	0.94–1.06	0.88
ECOG PS	0.82	0.42–1.60	0.57
COPD	2.58	0.81–8.25	0.11
Histo-type	1.80	0.97–3.32	0.04

Cox proportional-hazard regression analysis.

The difference concerning HIF-1α expression intensity multiplied by percentage (histoscore) and cells positive percentage are displayed in Table 5 (61 samples available). A significant difference in the histoscore was found being higher in the bone metastases group, (*p* = 0.02). By contrast a non significant difference in terms of percentage of positive cells was detected.

**Table 5:** HIF expression, difference between subgroups

Visceral	Metastases	Associated bone metastases	*p*
Intensity × percentage	20 (10.0–37.5)	50 (20–109.0)	0.02°
Percentage of posit cell	35.6 ± 24.7	46.8 ± 26.7	0.12°°

Mann–Whitney *U* test° Values expressed as median and interquartile range.

Fisher’s test values expressed as mean and SD.°°

**Figure 2:**
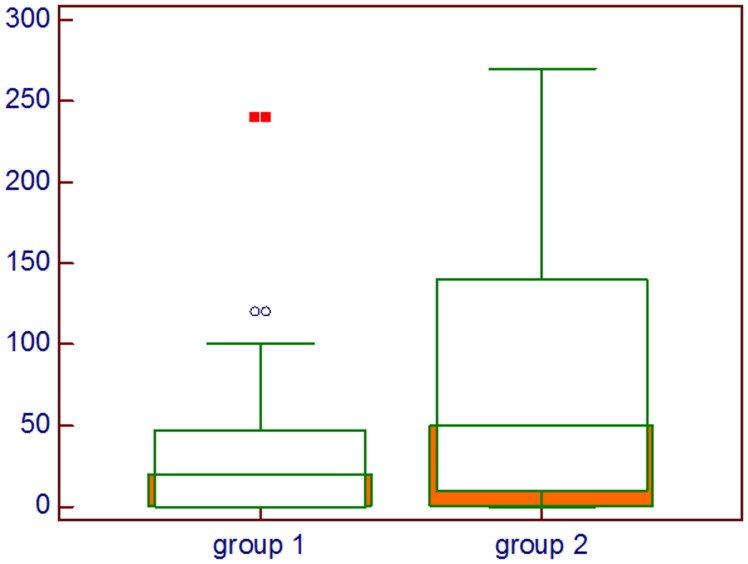
Comparison about HIF-1α intensity multiplied by percentage of positive cells; Group 1: Lung cancer with visceral metastases, Group 2: Lung cancer with bone and visceral metastases, Mann–Whitney *U* test.

**Figure 3:**
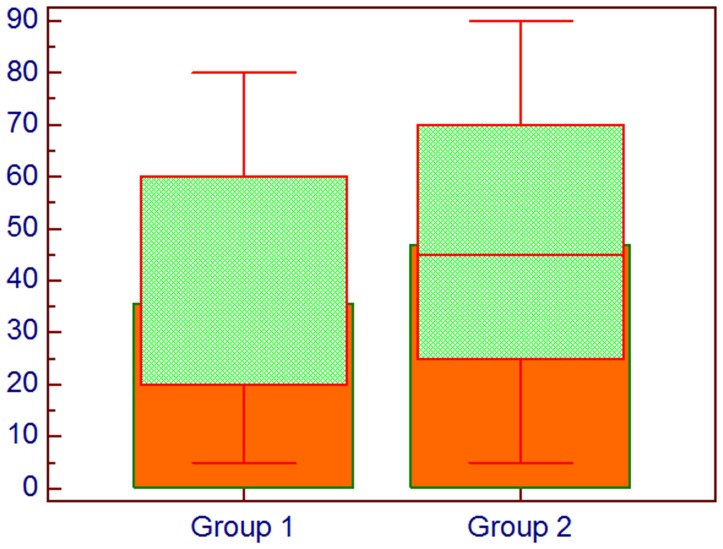
Comparison about percentage of cells expressing HIF-1α; Group 1: Lung cancer without bone metastases, Group 2: Lung cancer with bone metastases, Mann–Whitney *U* test.

In Figure 2 the difference between the groups about the histoscore of HIF-1α is shown, whereas in Figure 3 the difference in percentage of positive cells between groups is represented.

**Figure 4:**
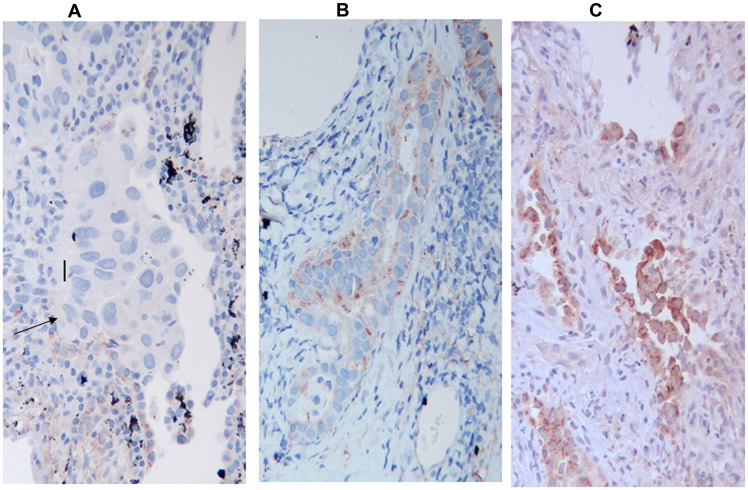
HIF-1α Immunostaining, (**A**) negative for HIF-1α, (**B**) mild positive, (**C**) high positive, The arrow indicates neoplastic cells. The figure indicates only the variation in the intensity of expression and not the percentage of positive cells.

A representation of the intensity staining for HIF-1α is depicted in Figure 4: a negative expression in Figure 4A, whilst a mild and high expression is represented in Figure 4B and 4C respectively.

## DISCUSSION

In the present study we have analyzed a sample of patients affected by metastatic non small cell lung cancer.

We focused on the comparison between patients with synchronous bone and visceral metastases and patients with only visceral metastases.

Not statistically significant differences regarding age, ECOG and comorbidities were detected.

The time to progression analysis displayed a significant difference with a clearly better survival in the group with visceral metastases compared to the group with bone metastases.

Our findings showed a close relationship between HIF expression and detection of bone metastases as expected. The combined higher intensity and percentage of positive cells for HIF expression is prevalent in patients with bone metastases. Our results confirm a role of HIF-1α in bone metabolism and its interactive function in bone microenvironment, according to different previous studies [15]. In fact, co-culture of monocytes with stromal cells including osteoblasts, fibroblasts, and cancer cells revealed that hypoxia-induced HIF-1α stimulates local production of pro-osteoclastogenic cytokines including receptor activator of nuclear factor kappa B ligand (RANKL), vascular endothelial growth factor (VEGF), inhibiting at the same time the production of osteoprotegerin (OPG), a soluble decoy receptor for RANKL that prevents osteoclasts maturation and activation [16, 17]. Furthermore, *in vitro* and *in vivo* studies demonstrated that adaptation to hypoxia is a critical step in tumor progression and it is regulated by HIF-1α. Hypoxia and HIF-1α were recognized to be responsible for enhanced osteolytic bone metastases in MDA-MB-231 breast cancer cell lines, causing a poor prognoses and an increased patient mortality [16].

Our study showed that HIF-1α predicts poor prognosis since it correlates with bone metastasis which is in turn associated with shorter time to progression.

It is involved in a wide spectrum of metabolic pathways leading to cancer cell activation and proliferation. We know from the literature that activated HIF-1α is able to stimulate the expression of several growth factors and glycolytic enzymes in human cancer cells [18]. Moreover, glucose is the main source for osteoclast action leading to bone resorption. The activation of genes encoding GLUT-1 and GLUT-2 was reported to implement the cancer cell metabolism favoring proliferation as well as the induction of mitochondrial reactive oxygen species (ROS) [19] and HIF-1 induces upregulation of GLUT-1 [20]. Those findings support our data suggesting that hypoxia is a frequent occurrence in bone metastatic tissue and it is able to promote by itself tumor cell proliferation and migration by the induction of several genes involved in cancer cell metabolism.

HIF is indeed a catalyst for tumor progression fostering several pathways such as epithelial-mesenchymal transition (EMT), characterized by changes in cell morphology and cell-matrix adhesion with loss of E-cadherin and overexpression of fibronectin and vimentin fostering cellular detachment [21].

High smoking exposition is associated with HIF-1α expression, as we can see in the present study. The synergic action of smoke compounds and hypoxia could foster HIF-1α expression by mithocondrial reactive oxygen species (ROS) [22, 23].

The present study points out other findings such as a relationship between smoking habit, pack-year index and HIF expression.

As far as the histotype is concerned, we know from the literature that squamous cell lung cancer is mainly associated with HIF expression [24].

Conversely, in the present study we found that adenocarcinoma was the prevalent histotype influencing the time to progression.

Aside from metabolic and genetic pathways activated by HIF, another aspect could explain its role in cancer progression. There is a relationship between HIF-1α and immune system.

Several mechanisms are responsible of HIF-1 induced tumor growth among which the resistance to T cell–mediated killing by increasing the expression of programmed death–ligand 1 (PD-L1) on tumor cells and the increased expression of CTLA-4 on CD8+ T cells. Further mechanisms includes the stabilization of NF-kB and the regulation of anti-apoptotic factors such as Bax, Bcl-2 during chemotherapy [25, 26].

## MATERIALS AND METHODS

From November 2015 to December 2016 we collected data of 146 patients with primary metastatic NSCLC. Among those 95 patients whose diagnosis was obtained by trans-thoracic or bronchial biopsy were selected in a retrospective analysis. The inclusion criteria were: Patients age ≥ 18 years old, former or current smoker with advanced non small cell lung cancer, available histology sample and detailed histotype. The exclusion criteria were lung cancer stage I-II-IIIA and patients with histological sample not available.

The staging of the disease was obtained by a total body computed tomography (CT) with contrast medium and confirmed by either 99-Tc scintigraphy if they were osteoblastic lesions or F18-FDG positron emission tomography if they were osteolytic. The time to progression was defined as the time elapsed from diagnosis until disease progression. All measurable lesions up to 2 lesions per organ were identified as target lesions with longest diameter of at least 10 mm. The progression disease was defined as a 20% increase in the sum of diameters of target measurable lesions, according to RECIST criteria version 1.1 [27].

The study was approved by Campus Bio-Medico di Roma Ethic Committee.

### Sample size and population

The sample size was determined by comparison of proportions with 80% power to detect HIF-1 α and the α value set at 0.05 of significance. At least overall 50 patients had to be recruited for this purpose.

The initial evaluation was carried out on a sample of 95 patients diagnosed with advanced lung cancer current or former smoker referred to the clinic. Two subgroups were identified according to bone metastases occurrence.

Ninety-five were the total population with proven positive histology obtained by trans-thoracic CT guided biopsies. Sixty-one of 95 cases had tumor blocks available for immunohistochemical analysis for HIF1α expression.

### Measurements and parameters

#### Demographic and functional baseline parameters

General demographic characteristics were collected, such as gender, age, smoking habit (never, previously, current), body mass index (BMI), smoking index risk defined as pack / year in order to distinguish between heavy and light smokers.

The historical and functional parameters collected were the following: performance status, comorbidities, staging by CT scan total body, FDG-PET/CT and optionally skeletal scintigraphy.

The histology tissue used for biological parameters detection derives from lung and bronchial biopsies.

### Techniques for histology specimen

Immunohistochemistry was performed on formalin-fixed paraffin-embedded sections of bronchoscopic or computer tomography-guided needle specimens. Representative paraffin blocks were cut into 3 μm sections that were mounted onto coated slides.

Afterwards, the sections were dewaxed by xylene and ethyl alcohol and rehydrated. All tissue sections slides were heated in citrate buffer solution at pH 6.0 for 40 minutes at 97° C, and then rinsed with H2O2 for 30 minutes.

Hence the sections were incubated with mouse monoclonal antibody anti-HIF-1α (clone H1 alpha 67; cat. no. NB100-105; Novus Biologicals, Littleton, CO, USA; 1:50) for 2 hours at room temperature.

After washing, the slides were incubated with secondary antibody conjugated with 2nd generation visualization kit which is suitable for both rabbit and mouse primary antibodies (DAKO), and the binding was displayed by 3-3′ diaminobenzine tetrahydrochloride after 30 minutes.

The staining results were scored semi-quantitatively as intensity: negative 0, mild 1, moderate 2 and high 3 and as percentage of positive cells. A histoscore was generated multiplying the intensity value (score 0–3) by the percentage of cells according to previous immune-histochemistry score evaluation studies [28].

### Statistical analysis

Data were expressed as mean ± standard deviation and median plus interquartile range as appropriate, with significance level set at *p* < 0.05.

The Fisher’s exact test was applied for categorical variables. The Mann Whitney test was applied for continuous variables not following a normal distribution, in order to detect differences between groups.

A multivariate logistic regression method was performed to detect the variables affecting HIF expression concerning percentage and intensity. A Kaplan-Meier analysis with log-rank test was applied to compare the TTP of the subgroups. The group available for the above test consists of 95 subjects.

A cox-proportional hazard regression analysis was performed to highlight which variables may affect the TTP as outcome.

The SPSS 24.0 statistical software package was used for analysis (Inc, Chicago, IL, USA).

## CONCLUSIONS

The study displays that HIF-1α expression is closely linked with the advanced disease and bone metastases occurrence. Patients with bone metastasis have a shorter time to progression than patients without it. Our findings support HIF-1α as a potential biomarker of bone metastasis and an indicator of poor prognosis in lung cancer.

## References

[1] Soni S, Padwad YS HIF-1 in cancer therapy: two decade long story of a transcription factor. Acta Oncol. 2017; 56:503–515. 10.1080/0284186X.2017.1301680. 28358664

[2] Liao D, Corle C, Seagroves TN, Johnson RS Hypoxia-inducible factor-1alpha is a key regulator of metastasis in a transgenic model of cancer initiation and progression. Cancer Res. 2007; 67:563–572. 10.1158/0008-5472.CAN-06-2701. 17234764

[3] Muz B, de la Puente P, Azab F, Azab AK The role of hypoxia in cancer progression, angiogenesis, metastasis, and resistance to therapy. Hypoxia (Auckl). 2015; 3:83–92. 10.2147/HP.S93413. 27774485 PMC5045092

[4] Rankin EB, Giaccia AJ Hypoxic control of metastasis. Science. 2016; 352:175–80. 10.1126/science.aaf4405. 27124451 PMC4898055

[5] Ke Q, Costa M Hypoxia-inducible factor-1 (HIF-1). Mol Pharmacol. 2006; 70:1469–1480. 10.1124/mol.106.027029. 16887934

[6] Bendinelli P, Maroni P, Matteucci E, Desiderio MA Cell and Signal Components of the Microenvironment of Bone Metastasis Are Affected by Hypoxia. Int J Mol Sci. 2016; 17. 10.3390/ijms17050706. 27187355 PMC4881528

[7] Tamura T, Kurishima K, Nakazawa K, Kagohashi K, Ishikawa H, Satoh H, Hizawa N Specific organ metastases and survival in metastatic non- small-cell lung cancer. Mol Clin Oncol. 2015; 3:217–221. 10.3892/mco.2014.410. 25469298 PMC4251107

[8] Daijo H, Daijo H, Hoshino Y, Kai S, Suzuki K, Nishi K, Matsuo Y, Harada H, Hirota K Cigarette smoke reversibly activates hypoxia-inducible factor 1 in a reactive oxygen species-dependent manner. Sci Rep. 2016; 6:34424. 10.1038/srep34424. 27680676 PMC5041075

[9] Zhu BQ, Heeschen C, Sievers RE, Karliner JS, Parmley WW, Glantz SA, Cooke JP Second hand smoke stimulates tumor angiogenesis and growth. Cancer Cell. 2003; 4:191–196. 10.1016/S1535-6108(03)00219-8. 14522253

[10] Tonini G, D’Onofrio L, Dell’Aquila E, Pezzuto A New molecular insights in tobacco-induced lung cancer. Future Oncol. 2013; 9:649–655. 10.2217/fon.13.32. 23647294

[11] Yoon V, Maalouf NM, Sakhaee K The effects of smoking on bone metabolism. Osteoporos Int. 2012; 23:2081–2092. 10.1007/s00198-012-1940-y. 22349964

[12] Wang X, Adjei AA Lung cancer and metastasis: new opportunities and challenges. Cancer Metastasis Rev. 2015; 34:169–171. 10.1007/s10555-015-9562-4. 25956388

[13] Siegel R, Naishadham D, Jemal A Cancer statistics, 2012. CA Cancer J Clin. 2012; 62:10–29. 10.3322/caac.20138. 22237781

[14] Hoffman PC, Mauer AM, Vokes EE Lung cancer. Lancet. 2000; 355:479–485. 10.1016/S0140-6736(00)82038-3. 10841143

[15] Knowles HJ Hypoxic regulation of osteoclast differentiation and bone resorption activity. Hypoxia (Auckl). 2015; 3:73–82. 10.2147/HP.S95960. 27774484 PMC5045091

[16] Hiraga T, Kizaka-Kondoh S, Hirota K, Hiraoka M, Yoneda T Hypoxia and hypoxia-inducible factor-1 expression enhance osteolytic bone metastases of breast cancer. Cancer Res. 2007; 67:4157–4163. 10.1158/0008-5472.CAN-06-2355. 17483326

[17] Guise TA, Mohammad KS, Clines G, Stebbins EG, Wong DH, Higgins LS, Vessella R, Corey E, Padalecki S, Suva L, Chirgwin JM. Basic mechanisms responsible for osteolytic and osteoblastic bone metastases. Clin Cancer Res. 2006; 12:6213s–6216s. 10.1158/1078-0432.CCR-06-1007. 17062703

[18] Suzuki N, Gradin K, Poellinger L, Yamamoto M Regulation of hypoxia-inducible gene expression after HIF activation. Exp Cell Res. 2017; 356:182–186. 10.1016/j.yexcr.2017.03.013. 28286304

[19] Semenza GL HIF-1: upstream and downstream of cancer metabolism. Curr Opin Genet Dev. 2010; 20:51–6. 10.1016/j.gde.2009.10.009. 19942427 PMC2822127

[20] Fan R, Hou WJ, Zhao YJ, Liu SL, Qiu XS, Wang EH, Wu GP Overexpression of HPV16 E6/E7 mediated HIF-1α upregulation of GLUT1 expression in lung cancer cells. Tumour Biol. 2016; 37:4655–4663. 10.1007/s13277-015-4221-5. 26508030

[21] Yang MH, Wu KJ TWIST activation by hypoxia inducible factor-1 (HIF-1): implications in metastasis and development. Cell Cycle. 2008; 7:2090–2096. 10.4161/cc.7.14.6324. 18635960

[22] Schaal C, Chellappan SP Nicotine-mediated cell proliferation and tumor progression in smoking-related cancers. Mol Cancer Res. 2014; 12:14–23. 10.1158/1541-7786.MCR-13-0541. 24398389 PMC3915512

[23] Guo L, Li L, Wang W, Pan Z, Zhou Q, Wu Z Mitochondrial reactive oxygen species mediates nicotine-induced hypoxia-inducible factor-1α expression in human non-small cell lung cancer cells. Biochim Biophys Acta. 2012; 1822:852–861. 10.1016/j.bbadis.2012.02.004. 22349311

[24] Takasaki C, Kobayashi M, Ishibashi H, Akashi T, Okubo K Expression of hypoxia-inducible factor-1α affects tumor proliferation and antiapoptosis in surgically resected lung cancer. Mol Clin Oncol. 2016; 5:295–300. 10.3892/mco.2016.937. 27446567 PMC4950225

[25] Pezzuto A, Carico E Role of HIF-1 in Cancer Progression: Novel Insights. A Review. Curr Mol Med. 2018; 18:343–351. 10.2174/1566524018666181109121849. 30411685

[26] Rohwer N, Cramer T Hypoxia-mediated drug resistance: novel insights on the functional interaction of HIFs and cell death pathways. Drug Resist Updat. 2011; 14:191–201. 10.1016/j.drup.2011.03.001. 21466972

[27] Eisenhauer EA, Therasse P, Bogaerts J, Schwartz LH, Sargent D, Ford R, Dancey J, Arbuck S, Gwyther S, Mooney M, Rubinstein L, Shankar L, Dodd L, et al New response evaluation criteria in solid tumors: revised RECIST giodeline (version 1.1). Eur J Cancer. 2009; 45:228–247. 10.1016/j.ejca.2008.10.026. 19097774

[28] Fedchenko N, Reifenrath J Different approaches for interpretation and reporting of immunohistochemistry analysis results in the bone tissue - a review. Diagn Pathol. 2014; 9:221. 10.1186/s13000-014-0221-9. 25432701 PMC4260254

